# Characterization of Unknown Orthobunya-Like Viruses from India

**DOI:** 10.3390/v10090451

**Published:** 2018-08-24

**Authors:** Shannon L. M. Whitmer, Pragya D. Yadav, Prasad Sarkale, Gouri Y. Chaubal, Alicia Francis, John Klena, Stuart T. Nichol, Ute Ströher, Devendra T. Mourya

**Affiliations:** 1 Viral Special Pathogens Branch, Centers for Disease Control and Prevention, Atlanta, GA 30333, USA; evk3@cdc.gov (S.L.M.W.); irc4@cdc.gov (J.K.); stn1@cdc.gov (S.T.N.); ute.stroeher@gmail.com (U.S.); 2 National Institute of Virology, Pune 411021, India; hellopragya22@gmail.com (P.D.Y.); prasadsarkale123@rediffmail.com (P.S.); gourimgupte@gmail.com (G.Y.C.); 3 School of Biology, Georgia Institute of Technology, Atlanta, GA 30322, USA; avfrancis14@gmail.com

**Keywords:** *Orthobunyavirus*, Cat Que virus, Manzanilla virus, pathogen discovery

## Abstract

Next-generation sequencing (NGS) of agents causing idiopathic human diseases has been crucial in the identification of novel viruses. This study describes the isolation and characterization of two novel orthobunyaviruses obtained from a jungle myna and a paddy bird from Karnataka State, India. Using an NGS approach, these isolates were classified as Cat Que and Balagodu viruses belonging to the Manzanilla clade of the Simbu serogroup. Closely related viruses in the Manzanilla clade have been isolated from mosquitos, humans, birds, and pigs across a wide geographic region. Since Orthobunyaviruses exhibit high reassortment frequency and can cause acute, self-limiting febrile illness, these data suggest that human and livestock infections of the Oya/Cat Que/Manzanilla virus may be more widespread and/or under-reported than anticipated. It therefore becomes imperative to identify novel and unknown viruses in order to understand their role in human and animal pathogenesis. The current study is a step forward in this regard and would act as a prototype method for isolation, identification and detection of several other emerging viruses.

## 1. Introduction

Identifying the etiology of disease has been an enduring challenge for microbiologists. First defined by Koch’s postulates, this axiom has experienced several modifications to incorporate new discoveries, such as viruses [1], and new technologies, such as molecular biology and next generation sequencing (NGS) [2,3]. NGS of agents causing idiopathic human diseases has been crucial for identifying isolated cases of obscure viral infections [4,5,6,7]. Herein, we describe the isolation and characterization of unknown infectious agents originally obtained from a jungle myna and paddy bird [8] from Karnataka State, India during the 1960s as part of surveys for the presence of Kyasanur Forest disease virus (KFDV), which had been recently identified within the same geographic region [9,10]. Since birds can serve as a zoonotic reservoir for human viruses, such as influenza and arboviruses [11,12], we performed additional characterization of these KFDV-negative bird specimens which identified several potential infectious agents, including an orthobunya-like virus.

The *Bunyavirales* order constitutes more than 350 viruses with tripartite negative- (or ambi-) sense, single-stranded RNA genomes belonging to 9 viral families [13]. Members of the *Feraviridae*, *Fimoviridae*, *Jonviridae*, *Nairoviridae*, *Peribunyaviridae*, *Phasmaviridae*, *Phenuiviridae*, and *Tospoviridae* families are carried and transmitted by arthropods, but viruses of the *Hantaviridae* family are carried by rodents and transmitted by contact with aerosolized excreta [13]. Viruses in the *Fimoviridae* and *Tospoviridae* families infect plants, while viruses in the *Nairoviridae*, *Peribunyaviridae*, *Phenuiviridae* families can infect vertebrates and exist in a zoonotic infection cycle—transmitting between humans and livestock via an arthropod intermediate.

Human infections caused by viruses of genus *Orthobunyavirus* can result in acute, self-limiting febrile illness (Oropouche virus [14]), or encephalitis (California encephalitis virus or La Crosse virus [15,16,17]). Symptoms of Oropouche virus infection are mild and can be misdiagnosed due to similarity with dengue, chikungunya, yellow fever or malaria [18,19], suggesting that infections by related viruses may also be misdiagnosed. Furthermore, Akabane, Aino and Schmallenberg viruses can also cause asymptomatic, or mild infection or congenital malformations in ruminants [20]. Thus, infections with orthobunyaviruses may be far more frequent and/or widespread than expected, and therefore, thorough analysis of infectious agents from human and animal samples is needed to truly understand the prevalence of these agents.

The International Committee on Taxonomy of Viruses (ICTV) defines the assignation of bunyaviruses into specific genera and species based on serologic cross-reactivity, protein and genome segment size, mechanism of genome expression (negative- or ambi-sense), and conserved terminal nucleotide sequences [21]. However, these criteria are malleable, and may change as novel bunyaviruses are discovered, or as phylogenetic relationships are further inferred. The incidence of rampant reassortment among bunyaviruses [22,23,24,25,26] with major phenotypic changes has resulted in difficulty in classification of related viruses. It therefore becomes necessary to determine the full genome sequences of these viruses in order to enable proper classification and understand their relevance to animal and human health.

## 2. Materials and Methods

### 2.1. NGS of Unknown Infectious Agents

At the National Institute of Virology (NIV), in Pune, India, suckling Swiss albino mice were inoculated intracranially with serum collected from a jungle myna bird (*Acridotheres fuscus*) in 1961 in the Sagar district of Karnataka State, India and mouse brain extract was grown in rhabdomyosarcoma (RD) cells at NIV and material was passaged in Vero E6 cells at The US Centers for Disease Control and Prevention (CDC). RNA was extracted from 100 µL of NIV tissue culture fluid and tissue culture cells using MagMAX Pathogen RNA/DNA isolation kit (Invitrogen, Carlsbad, CA, USA) and BeadRetriever (Invitrogen, Carlsbad, CA, USA), and 1mL of CDC viral isolates (tissue culture fluid and monolayer) using Tripure (Roche, Mannheim, Germany) followed by RNeasy Mini Kit to concentrate viral RNA to 20 µL (Qiagen, Hilden, Germany). The extracted viral RNA was treated with recombinant DNase I (RNase-free) (Roche, Mannheim, Germany) and ribosomal and carrier RNA were removed with complementary DNA oligonucleotides and RNase H as previously described [27,28]. RNA was reverse-transcribed using random hexamers and cDNA libraries were prepared using a Nextera XT DNA library preparation kit (Illumina, San Diego, CA, USA) as previously described [27,29]. DNA libraries were quantified using the High Sensitivity DNA Tapestation (Agilent, Santa Clara, CA, USA) and Kapa Library Universal Quantification kit (Kapa Biosystems, Wilmington, MA, USA). Libraries were pooled (original material 7-plex with other pathogen discovery specimens and CDC viral isolates 3-plex of JM1 tissue culture fluid, tissue culture cells, and mock infection control) and paired end sequenced using an Illumina MiSeq (version 2, 2 × 150 or 2 × 250 cycles). Sequencing of original material yielded 2.4 × 10^6^ and 16.5 × 10^6^ reads from the tissue culture fluid and cells, whereas sequencing of CDC viral isolates yielded 15.5 × 10^6^, 14.1 × 10^6^, and 3.6 × 10^6^ reads, respectively, from passage of the original tissue culture fluid, cells and mock-infected control. Average quality score was 36.46 ± 1.09 and >92.7 ± 4.3% of bases had confidence values greater than 30.

### 2.2. Bioinformatic Analysis

Pathogen discovery analysis of NGS reads was performed using EDGE v2.0.0. [30]. Reads were quality trimmed and filtered using default settings (trim quality level = 5, minimum read length = 50, “N” base cutoff = 10, low complexity filter = 0.85 average quality cutoff = 0; −min L 50 −avg. q 0 −n 2 −lc 0.85), de novo assembled into contigs and identified with blastx (NCBI-Bacteria-Virus) or raw reads were identified with gottcha (database version v20150825), metaphlan (v2.0.0), bwa (NCBI-Bacteria-Virus) and kraken_mini (v2.0.0). Orthobunya-like genomes were 5× iteratively assembled in Geneious (Geneious assembler, medium/low sensitivity) by mapping all orthobunyavirus-like reads identified by EDGE to different Manzanilla species (Table S2). Reads were assembled de novo using viral-ngs (v1.19.2) [31] and a custom Manzanilla-specific database. Classical swine fever virus (CSFV) genomes were 5× iteratively assembled in Geneious (Geneious assembler, medium/low sensitivity) by mapping all CSFV reads identified by EDGE to NC_002657. Additional sequence comparisons and trimming were performed in Geneious. Phylogenetic relationships of viruses of the Orthobunyavirus genus were inferred using translated sequences with phyml, v3.0 (-m LG -f m -a e -o tlr -s SPR). Node support was provided with 1000 bootstrap iterations. Phylogenetic trees were made using MEGA (v6.06). Cat Que and Balagodu genomes were deposited into Genbank: MH507151-6.

### 2.3. Reverse Transcription PCR and Sanger Sequencing

Reverse-transcription PCR (RT-PCR) was performed using SuperScript III One-step RT-PCR with Platinum Taq High Fidelity (Invitrogen, Carlsbad, CA, USA) with pathogen-specific primers (Table S3). Cycle conditions were: reverse transcription 30 min (1 cycle); 94 °C 15 s, Anneal 30 s, 68 °C 1 min/kb (40 cycles), 68 °C 5 min (1 cycle). JM1-specific primers were developed using the consensus viral sequence produced by NGS. Pestivirus-specific primers from Ridpath et al. amplifies all bovine viral diarrhea virus 1 (BVDV1) and bovine viral diarrheal virus 2 (BVDV2) isolates and classical swine fever virus (CSFV) [32]. Porcine endogenous retrovirus (PERV, also known as porcine type C oncovirus) protease-specific primers were from Patience et al. [32,33]. Mycoplasma specific primers were from van Kuppenveld et al. [33,34]. RT-PCR products were separated on 2% E-gels (Invitrogen) or using the D1000 ScreenTape and Tapestation (Agilent, Santa Clara, CA, USA). To perform Sanger sequencing, RT-PCR products were separated on 1% agarose gels, and specific bands of interest were excised and prepared for sequencing using a QiaQuick Gel Extraction kit (Qiagen, Hilden, Germany). DNA products were quantified and chain-termination PCR reactions were performed using pathogen-specific forward or reverse primers (Table S3) with the BigDye Terminator 3.1 sequencing kit (Applied Biosystems, Foster City, CA, USA). Cycle conditions were: 96 °C 1 min (1 cycle), 96 °C 10 s, 50 °C 15 s, 72 °C 1 min (40 cycles). BigDye reactions were cleaned using the DyeEx 2.0 spin kit (Qiagen, Hilden, Germany) or DyeEx 96 kit (Qiagen, Hilden, Germany). Purified chain-terminated reactions were sequenced using the ABI 3130XL genetic analyzer. Sequencing of low-passage PB1 (grown in *Vero CCL81* cells) was performed using pathogen-specific primers (Table S4) using Sanger sequencing. Products generated from Sanger sequencing were analyzed using Geneious (v10.1.3) and identified using Blastn [35]. 

### 2.4. Isolation and Characterization of Cat Que Virus Strains JM1 and PB1

At NIV serum from a jungle myna bird was initially used for intracranial inoculation (10 µL) of suckling Swiss albino mice (*n* = 8). Lyophilized filtered mouse brain suspension was resuspended in BAPS (2% BSA in phosphate buffered saline) and used for initial early attempts at viral propagation using African green monkey kidney (Vero CCL81), *Homo sapiens* muscle rhabdomyosarcoma (RD), and C6/36 mosquito cell lines. Mouse brain extract (10 µL) from the first passage was passaged between two and six additional times in suckling Swiss mice (8 mice per group), and lethality was established with serial dilutions using intracranial inoculations in all suckling mice. Mouse brain extract from the first passage (*n* = 3) and tissue culture fluid grown in RD cells (<3 passages procured from the NIV virus repository) (*n* = 7) were used to inoculate embryonated chicken eggs as described previously [36,37]. Estimated 8–10 days old embryonated eggs were inoculated with tissue culture fluid/mouse brain extract (200 µL per egg) by the allantoic route after surface decontamination of the eggs. The eggs were incubated at 35 °C in BOD incubator. The eggs were candled and lethality in the embryo was assessed every 24 h post infection till 7 days. Upon observation of lethality/hemorrhagic manifestation by candling, the embryos were harvested as described previously and the harvested embryos were observed for abnormality and hemorrhage. Balagodu virus, strain PB1 was collected from the serum of a paddy bird, *Ardeola grayii*, from Balagodu, Karnataka, India in 1963 [8]. All animal work was approved by the Indian Council of Medical Research (ICMR), National Institute of Virology (NIV) Animal Ethical Committee (IAEC registration number: 43/GO/ReBi/SL/99). JM1 and PB1 from lyophilized filtered mouse brain were propagated under biosafety level 2 conditions in Vero CCL81, PS and RD cells using MEM, supplemented with 10% fetal bovine serum (FBS), at 37 °C, 5% CO_2_. Cells were rinsed once with phosphate buffered saline (PBS) and 0.1 mL of the virus was added to the cells. Cells were incubated for 1 h at 37 °C with gentle agitation every 10 min. Inocula were removed and the cells were washed twice with PBS, culture medium was added to each well and cells were incubated at 37 °C, 5% CO_2_ for the duration of the experiment. Cell supernatant containing unknown cytopathic agent (JM1) (grown in RD cells, <3 passages procured from the NIV virus repository) was used in the current study. The unknown agent was sent to the US Centers for Disease Control and Prevention, and passaged once under biosafety level 4 conditions (due to suspected viral hemorrhagic fever virus) in Vero E6 cells grown in MEM supplemented with 2% FBS at 37 °C, 5% CO_2_ until cytopathic effect (CPE; change in media pH, vacuolation and spindling of adherent cells, and loss of monolayer) was observed at 3 days post infection.

## 3. Results

### 3.1. Identification of Novel Infectious Agents by Sequencing

**Figure 1:**
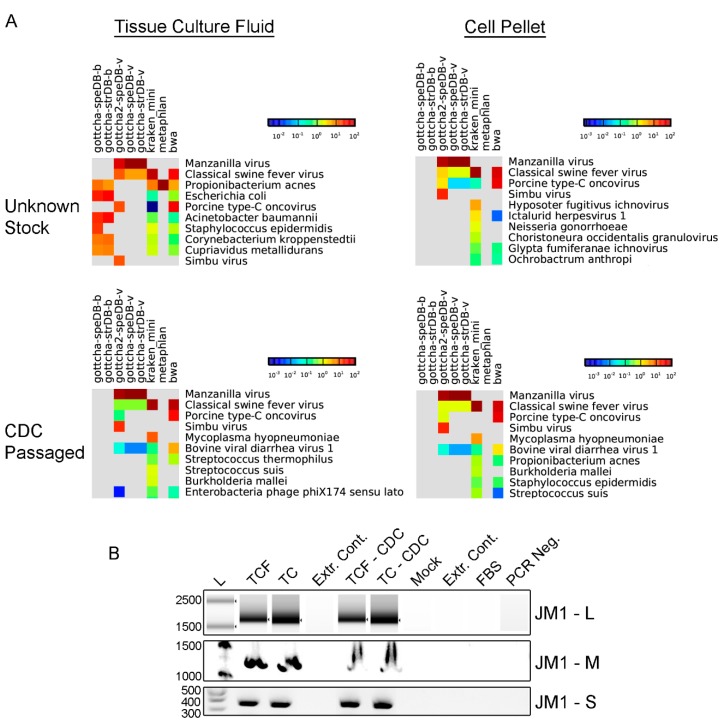
Identification of viral and bacterial nucleic acids using EDGE and RT-PCR from samples infected with JM1. (**A**) Identification of viral and bacterial reads using EDGE from specimens infected with JM1. Colors, as indicated by scale bar, show the number of reads specific to each organism. Only the top ten scoring organisms are presented. Columns present the results from individual pathogen identification programs (gottcha, gottcha2, ktaken, metaPhlan, bwa) and databases (speDB-b(acterial), speDB-v(iral)); (**B**) RT-PCR confirms presence of nucleic acids from JM1-infected samples. L—ladder, TCF—tissue culture fluid, TC—tissue culture monolayer, Extr. Cont.—Extraction control, FBS—Fetal bovine serum, PCR Neg.—PCR negative control. Images are compressed to remove extraneous lanes.

Suckling Swiss albino mice inoculated intracranially with serum collected from a jungle myna bird displayed signs of lethargy and sluggishness, suggesting the presence of an infectious agent. Mouse brain extract was grown in RD cells (<3 passages) and RNA was extracted from tissue culture fluid and the cell pellet and prepared for unbiased NGS to identify potential infectious agents. Analysis of NGS reads using the EDGE [30] system identified a complex mixture of viral and bacterial reads (Figure 1A). A high proportion of reads were identified as originating from Manzanilla/Simbu virus (>67% original material), and a minor fraction of reads were identified as CSFV (0.18%), and PERV (0.09%). Genome fold coverage for Manzanilla/Simbu virus from original material was 7818–6312-fold (Table S1). Re-passage of this material (CDC isolates) identified Manzanilla/Simbu virus as the dominant virus with a low numbers of reads (<1%) specific to CSFV, PERV, Mycoplasma hypopneumoniae, and BVDV (Figure 1A). Mock-infected cells did not contain reads originating from Manzanilla/Simbu virus, CSFV, and PERV, but did contain a low number of reads from *M. hypopneumoniae*, and BVDV (Figure S1), suggesting that passaging monolayers were the source of these reads.

A closer inspection of the viral and bacterial read counts identified that the majority of reads (>67% original material, and >77% CDC isolates) from these samples were from an orthobunya-like virus. To complete the viral genome, we assembled contigs from the CDC isolate samples using all orthobunya-like viral reads, resulting in 3328 contigs (200–2478 bp, average size = 241 bp, 90.3% of reads mapped to contigs). To avoid strain-specific assembly bias, we iteratively assembled contigs to Cat Que (JQ675598), Manzanilla (KP016012), Oya (JX983192), Ingwavuma (KF697139) or Mermet (KF697151) viruses (Table S2 and Figure S2). In general, the best assembly occurred using Manzanilla or Cat Que viruses as scaffolds and assembled genomes were nearly complete (M segment was missing 87 bases). A guided de novo assembly generated complete genomes (Figure S2). Viral consensus sequences assembled to Oya/Cat que/Manzanilla reference genomes or de novo assembled differed most in the extreme 3′ and 5′ ends, and trimming consensus genomes to scaffold genomes generated consensus sequences that exhibited 100% identity at the protein level (excluding regions with missing coverage in the M segment).

To confirm the presence of Cat Que virus RNA, RT-PCR and Sanger sequencing were performed using agent-specific primers. Primers recognizing each viral segment were generated using the JM1 consensus genome, and Sanger sequencing of the resulting PCR fragments confirmed the presence of the Cat Que genome in infected, but not mock-infected cells (Figure 1B and Table S3). To confirm the presence of RNA from potential cell culture contaminants, RT-PCR and Sanger sequencing were performed using pestivirus-specific and PERV-specific primers (Table S3) [32] and confirmed the presence of CSFV and PERV nucleic acids in infected cells, and BVDV in mock-infected cells and stock FBS used to generate tissue culture media (Figure S3A–C). Mycoplasma-specific bands were not detected in any sample.

The sequence for isolate PB1 was determined using primers specific for Cat Que and Ingwavuma viruses (Table S4).

### 3.2. Isolation and Characterization of an Unknown Infectious Agent

**Figure 2:**
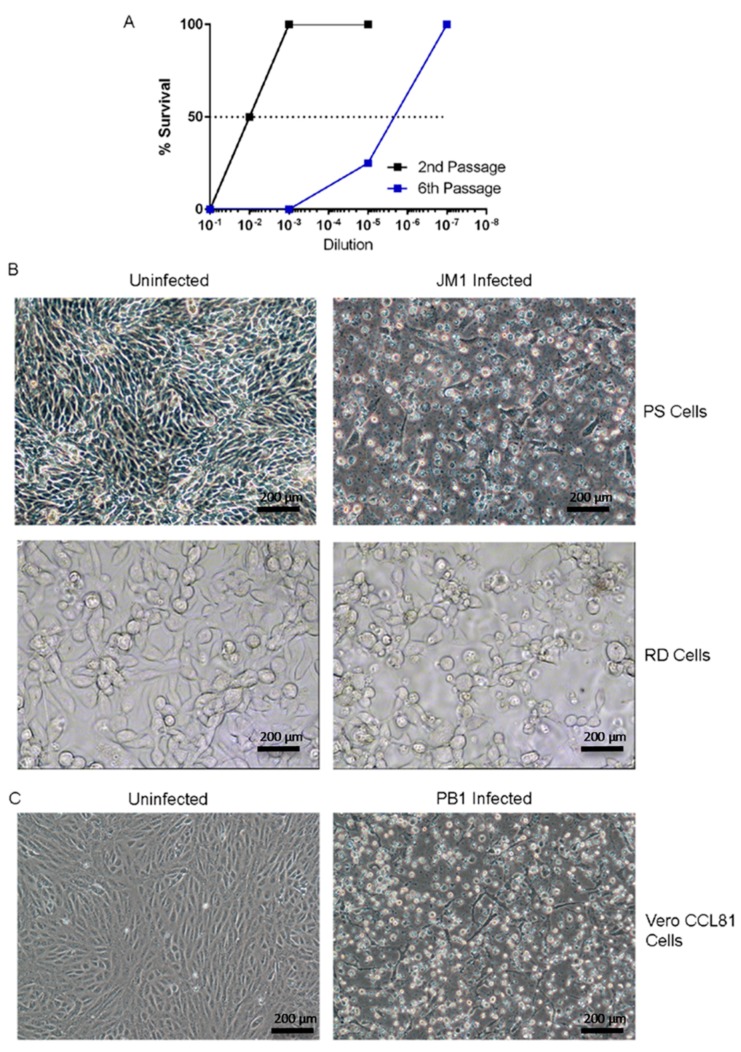
Isolation and characterization of novel infectious agents. (**A**) Overview of infectivity of JM1 after 2nd and 6th serial passage within infant mice; (**B**) Cytopathic effect seen in JM1-infected human pig kidney epithelial cells (PS) at 3 days post infection (**top**) and in rhabdomyosarcoma (RD) cells at 5 days post infection (**bottom**); (**C**) Cytopathic effect in PB1-infected Vero CCL81 cells at 3 days post infection.

The JM1 viral isolate was generated by passaging mouse brain extracts through additional rounds in suckling Swiss mice and lethality was observed to increase with passage (Figure 2A). The RD and porcine (PS) cells were inoculated with JM1 mouse brain extract from the 6th passage and CPE was observed after 5 and 3 days of growth, respectively (Figure 2B). PB1 mouse brain extract was passaged in Vero CCL81 cells with CPE observed after 3 days post infection (Figure 2C). JM1 pathogenesis was determined using mice and chick embryos (Table 1). All 1 or 10 days old mice inoculated through intraperitoneal (IP) or intracranial (IC) routes, respectively, succumbed to disease. Mice infected through the IP route at 10 or 17 days old exhibited increased time to death and decreasing mortality (from 50% to 12.5%), indicating that mice were less susceptible to IP viral infection with increasing age (Table 1, top). A subset of older mice (3/16) inoculated IP also exhibited paralysis and two of these mice succumbed to disease. JM1 also caused disease in chick embryos; infection with the JM1 viral isolate (original mouse brain suspension) caused hemorrhages on head and body, resulting in 60% lethality (Table 1). Inoculation with tissue culture fluid grown in RD cells (<3 passages procured from the NIV virus repository) resulted in similar hemorrhages and mortality was reduced to 14% (Table 1).

### 3.3. Inference of Phylogenetic Relationships

**Figure 3:**
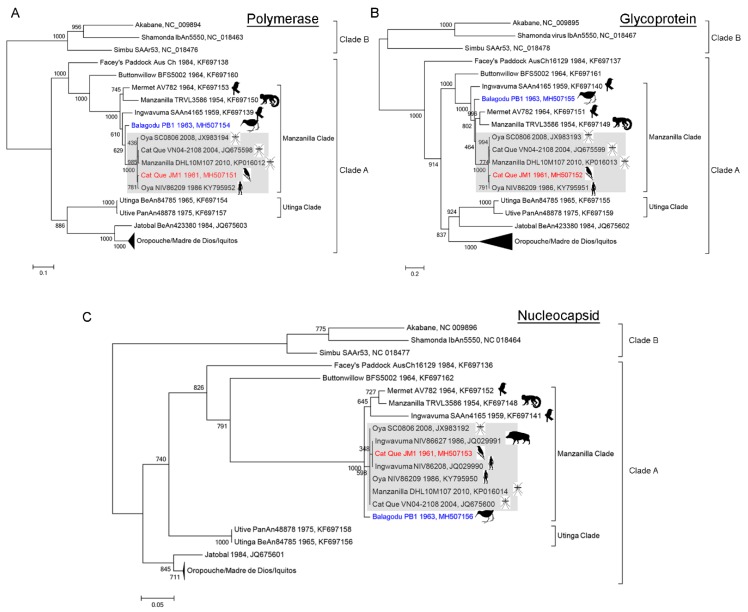
Phylogenetic analysis of members of the genus Orthobunyavirus, Simbu serogroup clade. New sequences included herein are highlighted in red (Cat Que JM1) and blue (Balagodu PB1). Images for organisms from which the viruses were originally isolated are included for the Manzanilla clade. The core Oya/Cat Que/Manzanilla subclade is shaded grey. (**A**) Phylogenetic relationships inferred using a protein alignment of RNA-dependent RNA polymerases from the L segment; (**B**) Phylogenetic relationships inferred using a protein alignment of full length glycoproteins from the M segment; (**C**) Phylogenetic relationships inferred using a protein alignment of nucleocapsid proteins from the S segment. Branch lengths are in substitutions/site.

To infer the relationship of the JM1 and PB1 orthobunya-like viruses, phylogenetic trees were built using protein sequences from the Simbu serogroup Clade A viruses, with three viruses from the Simbu serogroup Clade B included as an outgroup (Figure 3). For all proteins, the Simbu serogroup clade A and B structure was maintained. Within clade A the Oropouche, Utinga, Facey’s paddock, Buttonwillow and Manzanilla virus clades were also maintained. Within the Manzanilla clade, the inferred tree structure for the L, M, and S segments diverged into a “core” Oya/Cat Que/Manzanilla subclade (Figure 3A–C, grey) with amino acid identity of 96.2–99.6% (Supplemental Table S5) that was distinct from a Mermet/Manzanilla/Ingwavuma out group (78.5–95.7% similarity). JM1 (JM1, Figure 3A–C, red) consistently clustered within the core Oya/Cat Que/Manzanilla subclade (Figure 3A–C, grey). Nucleocapsid fragments collected from a pig (NIV86627) and a human (NIV86208) in India also clustered in this clade (Figure 3C, grey); despite their identification as Ingwavuma virus, they appear more closely related to Oya/Cat Que/Manzanilla virus. Manzanilla, strain DHL10M107 is also likely mis-identified and could be renamed to Oya or Cat Que virus. In contrast, PB1, collected from a paddy bird in India within the same time frame consistently clustered outside of the core Oya/Cat Que/Manzanilla subclade (Figure 3A–C, blue). PB1 was most closely related to a shared ancestor of the core Oya/Cat Que/Manzanilla subclade (polymerase and nucleocapsid, 92.8–98.2% identity, Supplemental Table S6) or shared ancestor of the Mermet/Manzanilla/Ingwavuma and Oya/Cat Que/Manzanilla subclades (glycoprotein, 83.1–86.8% identity). Since PB1 exhibits greater amino acid diversity than the core Oya/Cat Que/Mazanilla clade, yet is distinct from the Mermet/Manzanilla/Ingwavuma out group, we propose the unique name of Balagodu virus, from the origin of the collection.

## 4. Discussion

Here we describe the isolation and characterization of unknown infectious agents originally obtained from zoonotic samples collected in Karnataka, India during the 1960s as part of a survey for the presence of KFDV. Even though these specimens were collected in 1961 and 1963, they could not be characterized and identified at the time. Through NGS and using pathogen-specific oligonucleotides we identified an orthobunyavirus within the JM1 and PB1 samples. We established that JM1 could replicate and cause CPE within mammalian cells lines and that mice and chick embryos were susceptible to infection. Phylogenetic analysis inferred the evolutionary history of these viruses in relation to other orthobunyaviruses found in southeast Asia and worldwide.

Identifying and correlating the presence of an organism with disease has been a challenge for microbiologists since the time of Robert Koch (1890). Traditional pathogen discovery approaches, which have relied on culture, electron microscopy, and pathogen-specific PCR are problematic if the agent does not replicate. Novel or unexpected agents can also be easily missed if the organism does not cross-react with the applied diagnostic tests. Here we identified with an unbiased approach a previously uncharacterized infectious agent as Cat Que virus—this observation may have been missed with traditional single pathogen-specific diagnostic tools and demonstrates the sensitivity and specificity of virus isolation followed by NGS. However, this approach is not without pitfalls, as bioinformatic tools can mis-identify nucleic acids and additional minor contaminants can be identified. Mock-infected cell lines are critical to demonstrate whether passaging monolayers and cell media are the source of non-host reads (for example, the BVDV and mycoplasma-specific reads identified here—common tissue culture contaminants [38,39]). Here we observed that a major fraction of reads from infected cells were identified as Cat Que virus RNA and that the amount of Cat Que-specific reads increased with passage. In contrast, minor contaminant reads decreased (PERV and CSFV RNA) during passage suggesting a lack of significant replication. A remote possibility is that CSFV sequences found here (which were related to Indian CSFV strains isolated from pigs) may also have been originally isolated from the jungle myna bird or represent a species spill-over. However, in vitro studies with CSFV have established that birds within the *Corvus* genus cannot actively transmit the virus to pigs, nor do birds seroconvert following CSFV inoculation [40]. Birds within the *Acridotheres* genus may have different susceptibility to CSFV, or the potential source may be cross-contamination from a cell line or other external source. Because of the significant number of Cat Que RNA reads in the jungle myna bird samples that increased with passage, we hypothesize that this virus was likely originally carried by birds and not introduced from an external source during passaging.

**Figure 4:**
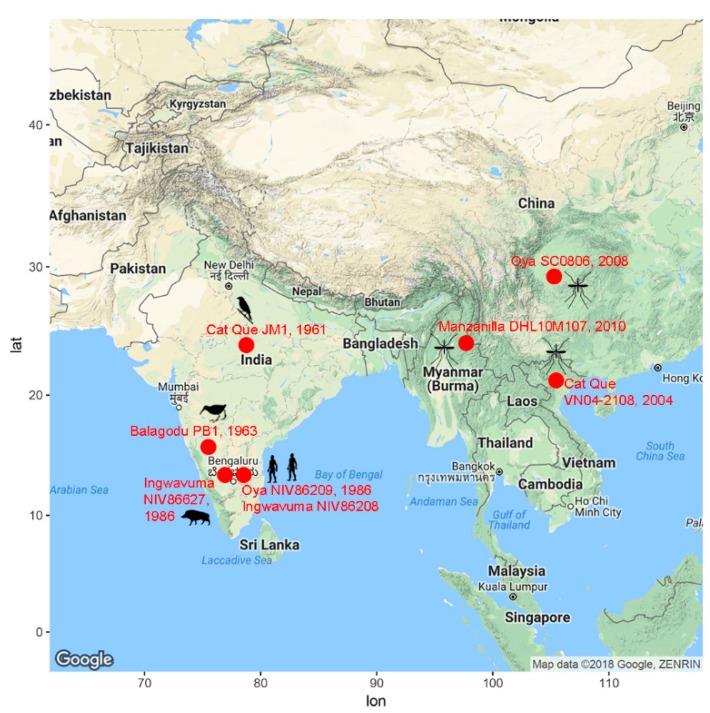
Geographic, temporal, and host distribution of Oya/Cat Que/Manzanilla virus subclade. Only sequences from the core Oya/Cat Que/Manzanilla subclade (Figure 4, shaded grey) are included in the map.

Viruses closely related to the core Oya/Cat Que/Manzanilla subclade (Figure 3, grey) have been isolated from mosquitos, humans, and pigs (Figure 4) [41,42,43,44].

Here, we demonstrate that viruses within this core clade can also be isolated from birds. Given the degree of genetic similarity of viruses within the core Oya/Cat Que/Manzanilla subclade, consistent viral nomenclature (Cat Que, strain JM1; Cat Que, strain SC0806) would simplify their phylogeny, as has previously been suggested by Ladner et al. [23]. The host prevalence of Oya/Cat Que/Manzanilla viruses mimics enzootic bird-mosquito and bird-mosquito-swine transmission observed for other arboviruses, such as West Nile and Japanese encephalitis viruses [45]. For most arboviruses, zoonotic spillover from an infected reservoir (pigs, birds, ruminants, mosquitos) population contributes to human outbreaks. To date, viruses within the Manzanilla clade have been isolated from only two human samples collected from India in 1986 (JQ029990 and KY795950) [41]. However, given the wide geographic, temporal and host range (Figure 4) of the Oya/Cat Que/Manzanilla subclade in Southeast Asia, and the fact that viruses in the Oropouche serocomplex can cause acute, non-specific febrile illness [19], these data suggest that Oya/Cat Que/Manzanilla human and livestock infections may be more widespread and/or under-reported than anticipated. Closely related Mermet, Manzanilla, and Ingwavuma viruses have been isolated from vertebrates in Mermet, Illinois, USA, Trinidad, and South Africa, respectively, demonstrating a potential worldwide prevalence for the Manzanilla clade. Developing serological and molecular diagnostic tests is necessary to understand the prevalence of this virus in Southeast Asia (and worldwide) and thereby correlate it with specific signs and symptoms of disease and any zoonotic contact.

While the prevalence of Manzanilla clade viruses is currently unknown, many factors could contribute to the emergence (or re-emergence) and spread of these arboviruses. Oya virus has been previously isolated from a wide range of mosquito species (*Culex*, *Anopheles* and *Masonia*) [44]. The dispersal of infected vector and zoonotic reservoir species, through deforestation, climate change, human activity, viral mutation and/or decline in prevalence of a competing viral species has previously contributed to the spread of Japanese encephalitis and West Nile viruses [45] and may also contribute to the emergence of hitherto unknown diseases. It therefore becomes imperative to identify novel and unknown viruses in order to understand their roles in human and animal pathogenesis. This paper describes an approach by which new viral agents can be identified and characterized. The current study is a step forward in this regard and presents a combination of traditional virological and NGS methods for the isolation, identification and detection of several emerging viruses.

## Figures and Tables

**Table 1:** JM1 virus pathogenesis in chick embryos and newborn mice.

Inoculum	Organism	Route	Age	Mortality	Mortality at Day Post Infection (DPI)	Remarks
Mouse brain suspension, Neat	Swiss mice	intraperitoneal	1 do	8/8	4	
Mouse brain suspension, Neat	Swiss mice	intracranial	10 do	8/8	3	
Mouse brain suspension, Neat	Swiss mice	intraperitoneal	10 do	4 */8	6	* Paralyzed Mice died on 7 dpi, Left leg paralysis noted in a survivor.
Mouse brain suspension, Neat	Swiss mice	intraperitoneal	17 do	1 */8	7	* Paralyzed mouse died on 8 dpi.
Mouse brain suspension, Neat	Chicken	Allantoic	est. 8–10 do	2/3	2, 3	Hemorrhages on head, frank oozing of blood from skull, generalized edema, body hemorrhages.
Tissue Culture Fluid (<3 passages)	Chicken	Allantoic	est. 8–10 do	1/7	6	Hemorrhages on heart and liver.

*: Mouse paralyzed prior to death.

## Supplementary Materials

The following are available online at http://www.mdpi.com/1999-4915/10/9/451/s1, Table S1: Summary of JM1 viral read prevalence and viral genome coverage. Percent refers to specific viral reads out of total reads; values in parentheses indicate numbers of specific viral reads and numbers of de-duplicated viral reads. Figure S1: Identification of viral and bacterial reads using EDGE from extraction control and mock-infected specimens. Colors, as indicated by the scale bar, show the number of reads specific to each organism. Only the top ten scoring organisms are presented. Columns present the results from individual pathogen identification programs (gottcha, gottcha2, ktaken, metaPhlan, bwa) and databases (speDB-b(acterial), speDB-v(iral)). Table S2: Assembly of orthobunya-like viral contigs to genomes comprising the Manzanilla clade. Figure S2: Overview of bioinformatic analysis. Table S3: Confirmatory virus- and bacterial-specific RT-PCR primers. Figure S3: Viral and bacterial-specific RT-PCR confirms presence of nucleic acids from JM1-infected samples. (A) RT-PCR using primers recognizing Pestivirus, including bovine viral diarrheal virus-1 (BVDV1), bovine viral diarrheal virus-2 (BVDV2), and classical swine fever virus (CSFV); (B) RT-PCR with primers recognizing porcine endogenous retrovirus (PERV, also called porcine type-C oncovirus); (C) RT-PCR using primers specific for Mycoplasma spp. L—ladder, TCF—tissue culture fluid, TC—tissue culture monolayer, Extr. Cont.—Extraction control, FBS—Fetal bovine serum, PCR Neg.—PCR negative control. Images are compressed to remove extraneous lanes. Table S4: Primer pairs used in RT-PCR reactions for full genome sequencing of PB1. Table S5: Comparison of JM1 versus Manzanilla and Utinga clade genomes. Values are presented as nucleotide or amino acid percent identity. Table S6: Comparison of PB1 versus Manzanilla and Utinga clade genomes. Values are presented as nucleotide or amino acid percent identity.
