# Supplementary figures and images for: Characterization of Unknown Orthobunya-Like Viruses from India
Source: Viruses. 2018 Aug 24;10(9):451. doi: 10.3390/v10090451 (PMC6165560; doi:10.3390/v10090451)

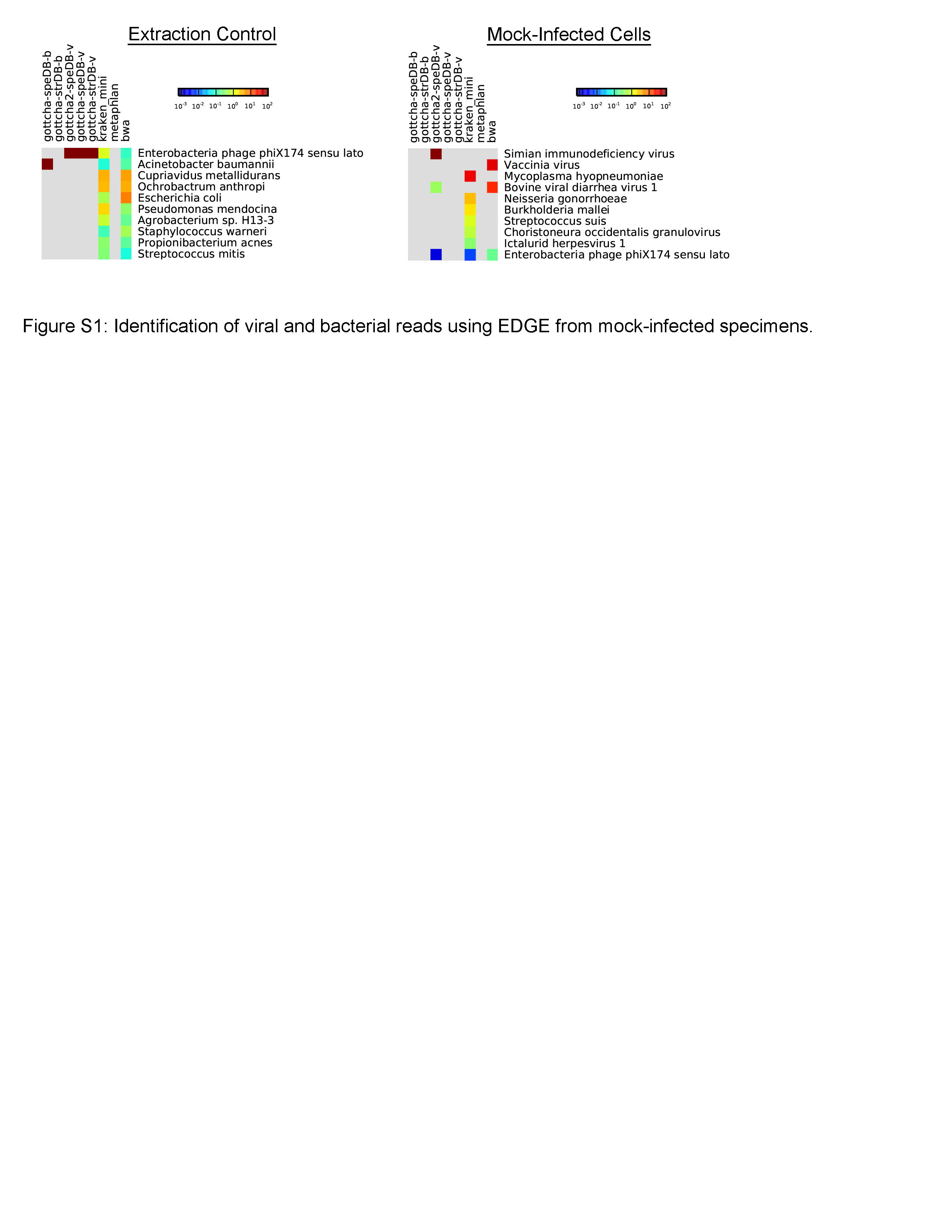

Supplement: Supplementary file 1 [file viruses-10-00451-s001.zip › Supplementary_Figure_1_V1.tif]

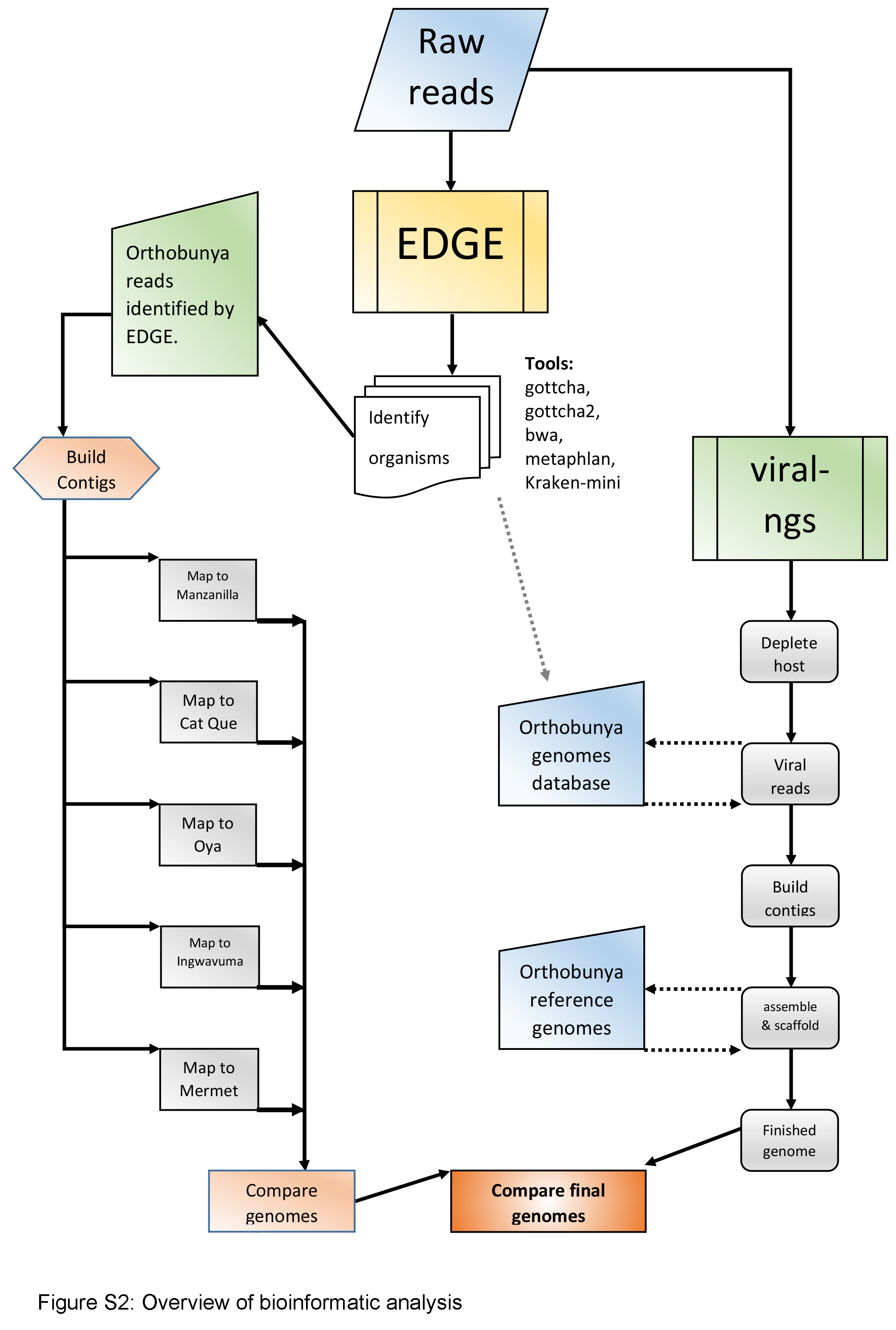

Supplement: Supplementary file 1 [file viruses-10-00451-s001.zip › Supplementary_Figure_2_V2.tif]

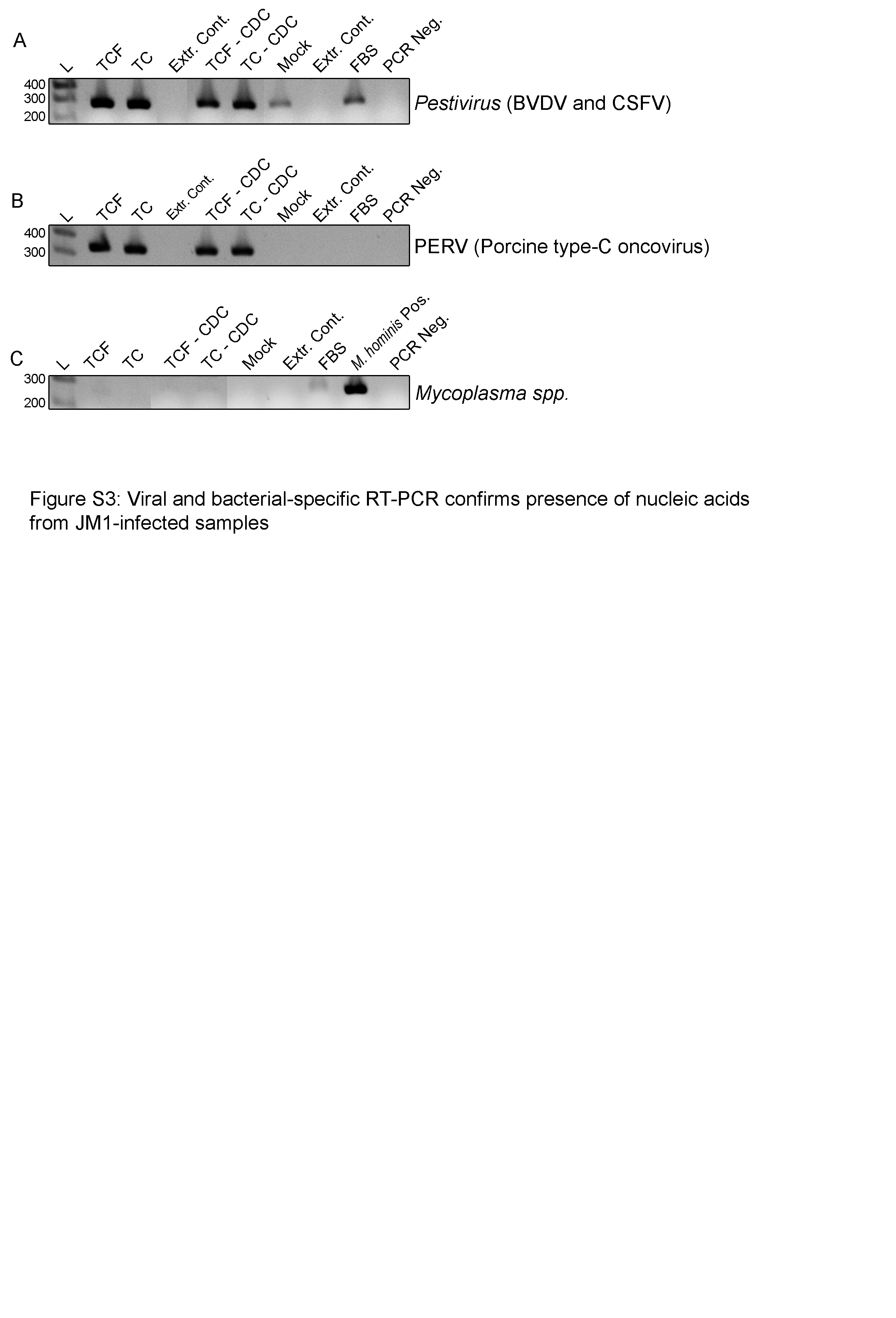

Supplement: Supplementary file 1 [file viruses-10-00451-s001.zip › Supplementary_Figure_3_V2.tif]
